# Detection and Identification of Probiotic *Lactobacillus plantarum* Strains by Multiplex PCR Using RAPD-Derived Primers

**DOI:** 10.3390/ijms161025141

**Published:** 2015-10-22

**Authors:** Alex Galanis, Yiannis Kourkoutas, Chrysoula C. Tassou, Nikos Chorianopoulos

**Affiliations:** 1 Department of Molecular Biology and Genetics, Democritus University of Thrace, Alexandroupolis 68100, Greece; E-Mail: ikourkou@mbg.duth.gr; 2 Institute of Technology of Agricultural Products, Hellenic Agricultural Organization-DEMETER, Sof. Venizelou 1, Lycovrissi, Attiki 14123, Greece; E-Mail: ctassou@nagref.gr

**Keywords:** probiotics, lactic acid bacteria, *Lactobacillus plantarum*, multiplex PCR, RAPD, SCAR

## Abstract

*Lactobacillus plantarum* 2035 and *Lactobacillus plantarum* ACA-DC 2640 are two lactic acid bacteria (LAB) strains that have been isolated from Feta cheese. Both display significant potential for the production of novel probiotic food products. The aim of the present study was the development of an accurate and efficient method for the molecular detection and identification of the above strains in a single reaction. A multiplex PCR assay was designed for each strain, based on specific primers derived from Random Amplified Polymorphic DNA (RAPD) Sequenced Characterized Amplified Region (SCAR) analysis. The specificity of the assay was tested with a total of 23 different LAB strains, for *L. plantarum* 2035 and *L. plantarum* ACA-DC 2640. The multiplex PCR assay was also successfully applied for the detection of the above cultures in yogurt samples prepared in our lab. The proposed methodology may be applied for monitoring the presence of these strains in food products, thus evaluating their probiotic character. Moreover, our strategy may be adapted for other novel LAB strains with probiotic potential, thus providing a powerful tool for molecular discrimination that could be invaluable to the food industry.

## 1. Introduction

Probiotics are living microorganisms, with bifidobacteria and lactic acid bacteria (LAB) constituting the most common microbial groups. According to the Food and Agriculture Organization (FAO) and the World Health Organization (WHO) of the United Nations, probiotics are defined as “live microorganisms which when administered in adequate amounts confer a health benefit on the host” [1]. The theory that regular consumption of lactic acid bacteria in fermented foods may contribute to enhancing health and longevity was originally developed by the Russian immunologist and Nobel Laureate in Medicine Elie Metchnikoff and was presented in his book “The prolongation of life”, published in 1907. Nowadays, probiotics represent a key fast growing section of the food industry and are added to a number of products on the market, including yogurts, cheeses, ice creams and other desserts [2]. According to a market report, probiotics’ demand worldwide was worth $27.9 billion in 2011 and is estimated to reach $44.9 billion in 2018, increasing at an annual growth rate of 6.8% from 2013 to 2018 [3]. Their strong market development and immense future potential are mainly due to numerous studies that have linked consumption of probiotic beverages, foods, and supplements to beneficial effects for consumers’ health [4]. Indeed, a probiotic-rich diet has been connected with the prevention and potential treatment of several digestive disorders, such as irritable bowel syndrome [5], necrotizing enterocolitis (NEC) in neonates [6], and ulcerative colitis [7]. Recent studies employing *in vitro* and *in vivo* systems have also indicated that probiotic treatment might reduce the risk for colon, liver, and bladder cancers [8]. It may also be effective against diet-induced obesity through the modulation of genes associated with metabolism and inflammation in the liver and adipose tissue [9], and even be therapeutically beneficial for the treatment of anxiety and depression [10].

It is evident that isolation and characterization of new LAB strains with probiotic potential attracts significant interest by the food industry for the development of novel functional food products. In this context, two *Lactobacillus plantarum* strains, termed as 2035 [11] and ACA-DC 2640 [12] were isolated from Feta cheese. Both exhibit probiotic potential and biotechnologically important characteristics, as demonstrated by several *in vitro* tests, such as bile salt tolerance, ability to grow at low pH, antimicrobial activity, antibiotic resistance *etc.* [12,13,14,15]. However, to further evaluate the properties of these strains and establish their probiotic character, verification of their presence and monitoring of their levels in food products are required. For this reason, an accurate method of strain detection and identification is needed.

Conventional microbiological tests for phenotypical characterization are considered inadequate, as they have limitations in discriminating large numbers of isolates with similar physiological characteristics. Several DNA-based techniques have been developed to overcome this obstacle [16]. For example, the Pulsed Field Gel Electrophoresis (PFGE) methodology is commonly used for probiotic strain differentiation and discrimination [17,18]. However, it cannot be applied for direct detection of a particular strain, in a single reaction [17]. Moreover, it is laborious, time-consuming and, thus, inappropriate for large scale screening experiments *in vivo*, especially when microbial groups, other than those needed to be identified, are at higher population levels. On the other hand, the multiplex PCR methodology is commonly employed for efficient single step detection of LAB strains in probiotic products [19,20,21]. It is easy to implement, fast, cost efficient, and requires a small amount of template DNA. Simultaneous amplification of two or more loci in the same reaction with strain specific primers that target the variable regions of the *16S rRNA*, the *hsp60* or other universal genes is performed, thus increasing the sensitivity and accuracy of discrimination. However, when sequence information of the target bacteria is not available, the design of specific primers by comparative sequence analysis is not feasible. To overcome this limitation, the random amplified polymorphic DNA (RAPD) technique may be applied. RAPD is a PCR-based assay that uses short arbitrary primers that anneal to multiple random target sequences to generate the needed polymorphism [22,23]. The revealed polymorphic sites, namely sequence characterized amplified regions (SCAR), are then used for the development of longer, sequence complementary primers. A more stringent annealing temperature than that used with the RAPD analysis is also applied for the novel primers, thus increasing the specificity and reproducibility of the assay.

The aim of the present study was the development of an assay for efficient and accurate molecular detection of *L. plantarum* strains 2035 and ACA-DC 2640 in a single reaction. RAPD SCAR analysis was firstly applied for the development of strain-specific primers as no sequence data was available for either of the two strains.

## 2. Results and Discussion

### 2.1. Screening of RAPD Primers and Isolation of SCAR Markers

**Figure 1:**
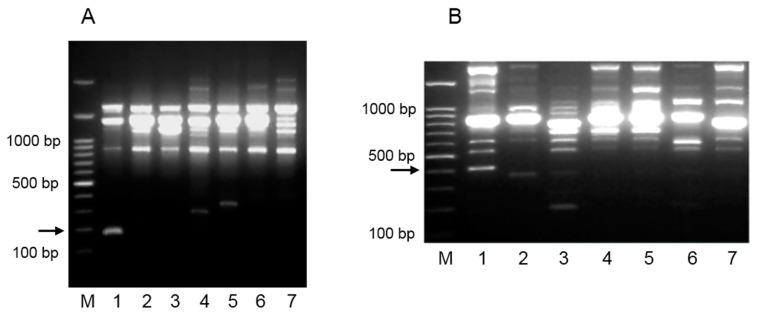
RAPD analysis for *L. plantarum* strains 2035 and ACA-DC 2640. (**Α**) Electrophoretic profile generated with RAPD primer RAPD-30, of *L. plantarum* 2035 (lane 1), and 6 *L. plantarum* wild type strains (lanes 2–7). Lanes: 2, *L. plantarum* 141; 3, *L. plantarum* E95; 4, *L. plantarum* E106B; 5, *L. plantarum* E128; 6, *L. plantarum* E89; 7, *L. plantarum* E119. M: 1 kb DNA ladder. The numbers on the left of the figure indicate the DNA size markers in base pairs (bp). The PCR product of 179 bp that was isolated from the agarose gel for further analysis is indicated with the arrow; (**Β**) Electrophoretic profile generated with RAPD primer RAPD-29, of *L. plantarum* ACA-DC 2640 (lane 1), and 6 *L. plantarum* wild type strains (lanes 2–7). Lanes: 2, *L. plantarum* 141; 3, *L. plantarum* E95; 4, *L. plantarum* E106B; 5, *L. plantarum* E128; 6, *L. plantarum* E89; 7, *L. plantarum* E119. M: 1 kb DNA ladder. The numbers on the left of the figure indicate the DNA size markers in base pairs (bp). The PCR product of 401 bp that was isolated from the agarose gel for further analysis is indicated with the arrow.

A total of 120 arbitrary primers were tested with RAPD PCR with DNA extracted from pure cultures of *L. plantarum* strains 2035 and ACA-DC 2640. Thirty-one primers for *L. plantarum* 2035 and 37 for *L. plantarum* ACA-DC 2640 produced more than four scorable bands and were chosen for further analysis. Out of the primers selected, primers RAPD-30 and RAPD-29 gave unique RAPD profiles for *L. plantarum* 2035 and *L. plantarum* ACA-DC 2640, respectively (Figure 1). The experimental procedure was repeated at least three times with the same conditions to confirm the reproducibility of the RAPD analysis. A PCR product of 179 bp for *L. plantarum* 2035 (Figure 1Α) was isolated from the agarose gel, cloned into an appropriate pBlueScript vector, and sequenced. Similarly, in the case of *L. plantarum* ACA-DC 2640, a 401 bp band was considered as potential strain-specific RAPD marker and isolated for further analysis (Figure 1Β).

### 2.2. RAPD-Derived Primers for L. plantarum 2035 and L. plantarum ACA-DC 2640

**Figure 2:**
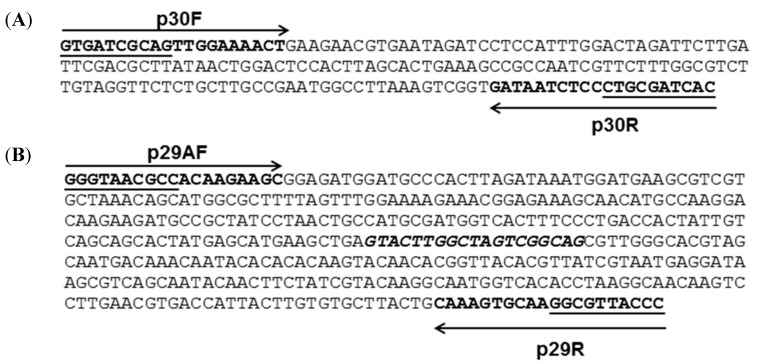
Nucleotide sequences of RAPD amplicons. (**A**) Nucleotide sequences of RAPD amplicons specific to *L. plantarum* 2035. Arrows represent forward primer p30F and reverse primer p30R and the underlined sequence belongs to the oligo decamer primer RAPD-30; (**Β**) Nucleotide sequences of RAPD amplicons specific to *L. plantarum* ACA-DC 2640. Arrows represent forward primer p29AF and reverse primer p29R and the underlined sequence belongs to the oligodecamer primer RAPD-29. The sequence of the forward primer p29BF is in bold italics.

The nucleotide sequences of the RAPD markers were used to design novel and potentially strain-specific primers for *L. plantarum* strains 2035 and ACA-DC 2640 (Figure 2). The nucleotide sequences for the primers of *L. plantarum* 2035 were: Forward primer (p30F): 5′-GTGATCGCAGTTGGAAAACTG-3′, and Reverse primer (p30R): 5′-GTGATCGCAGGGAGATTATC-3′. The primers were designed to the 5′ and 3′ ends of the obtained sequences (Figure 2Α). The nucleotide sequences for the primers of *L. plantarum* ACA-DC 2640 were: Forward primer A (p29AF): 5′-GGGTAACGCCACAAGAAGC-3′, Forward primer B (p29BF): 5′-GTACTTGGCTAGTCGGCAG-3′ and Reverse primer (p29R): 5′-GGGTAACGCCTTGCACTTTG-3′. The primers p29AF and p29R target the 5′ and 3′ ends of the RAPD marker, respectively, whereas the primer p29BF targets an internal potentially polymorphic region (Figure 2Β). A set of primers (Lac1F and Lac2R) that recognizes the *16S rRNA* gene of all *Lactobacillus* strains and generates a 340 bp product was also comprised as a positive control marker [24]. The specificity of the primers and the estimated size of PCR products are presented in Table 1. Optimization of the reactions was performed by adjusting the amount of all primers, the concentration of dNTPs and MgCl_2_, and the cycling conditions (extension and annealing time and temperature and number of cycles) (data not shown) [25].

**Table 1:** Specificity of primer pairs for Multiplex PCR on *L. plantarum* 2035 and *L. plantarum* ACA-DC 2640. The expected size of PCR products is indicated.

Reference Strain	Specificity of Primer Pairs			
	p30F/p30R (179 bp)	p29AF/p29R (401 bp)	p29BF/p29R (200 bp)	Lac1F/Lac1R (340 bp)
*L. plantarum* 2035	+	−	−	+
*L. plantarum* ACA-DC 2640	−	+	+	+

### 2.3. Multiplex PCR with RAPD-Derived Strain Specific Primers

**Figure 3:**
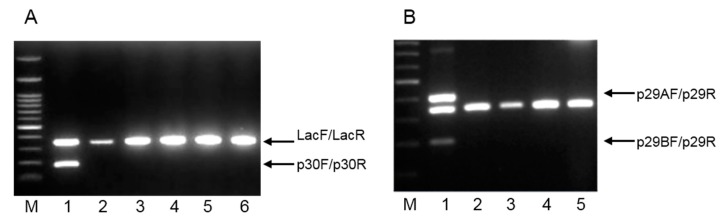
Agarose gel electrophoresis of PCR products from multiplex PCR assay. (**A**) Lanes: 1, *L. plantarum* 2035; 2, *L. plantarum* 141; 3, *L. plantarum* E95; 4, *L. plantarum* E106B; 5, *L. plantarum* E128; 6, *L. plantarum* E89. M: 1 kb DNA ladder. A PCR product of 179 bp was generated from the primer set p30F/p30R, whereas a PCR product of 340 bp was generated from the primer set Lac1F/Lac1R. Both products are indicated with the corresponding arrows; (**B**) Lanes: 1, *L. plantarum* ACA-DC 2640; 2, *L. plantarum* 141; 3, *L. plantarum* E95; 4, *L. plantarum* E106B; 5, *L. plantarum* E128. M: 1 kb DNA ladder. Two PCR products of 401 bp and 200 bp were generated from the primer sets p29AF/p29R and p29BF/p29R, respectively, and are indicated with the corresponding arrows. A PCR product of 340 bp was generated from the primer set Lac1F/Lac1R.

The multiplex PCR was initially tested with DNA extracted from *L. plantarum* 2035 and five other *L. plantarum* strains. As presented in Figure 3Α, *L. plantarum* 2035 gave two distinct products (179 and 340 bp), whereas only one, the 340 bp positive control product, was produced for the five other *L. plantarum* strains tested (Figure 3A). In a similar way, a multiplex PCR was performed with DNA from *L. plantarum* ACA-DC 2640 and four other *L. plantarum* strains. Significantly, only three PCR products were produced (401, 340 and 203 bp) for *L. plantarum* ACA-DC 2640 promoting the specificity of the reaction (Figure 3Β).

To further validate the specificity of the RAPD-derived primers for *L. plantarum* strains 2035 and ACA-DC 2640, the multiplex PCR assay was also tested with DNA extracted from 17 *L. plantarum* as well as from six other LAB strains, including the reference strains *L. casei* Shirota ACA-DC 6002, *L. rhamnosus* GG ATCC 53103 and *L.* *casei* ATCC 393, which are commonly used in probiotic products. Consistently, the primer pair p30F and p30R generated a distinct product only for *L. plantarum* 2035 (Table 2). Similarly, the specificity of the novel primers for *L. plantarum* ACA-DC 2640 was positively tested in a total of 23 LAB strains as indicated in Table 2.

**Table 2:** The complete list of *Lactobacillus* strains tested in multiplex PCR with the RAPD-derived primer pair p30R/p30F for *L. plantarum* 2035 and primer pairs p29AR/p29F and p29BR/p29F for *L. plantarum* ACA-DC 2640, respectively. A unique 2-band pattern is only produced for *L. plantarum* 2035 and a unique 3-band pattern is only produced for *L. plantarum* ACA-DC 2640 (indicated as +).

Strain Tested	2-Band Pattern	3-Band Pattern
*Lactobacillus plantarum* 2035	+	−
*L. plantarum* ACA-DC 2640	−	+
*L. plantarum* E1	−	−
*L. plantarum* E4	−	−
*L. plantarum* E45	−	−
*L. plantarum* E50	−	−
*L. plantarum* E66	−	−
*L. plantarum* E68	−	−
*L. plantarum* E71	−	−
*L. plantarum* E73	−	−
*L. plantarum* E77	−	−
*L. plantarum* E79	−	−
*L. plantarum* E10	−	−
*L. plantarum* E69	−	−
*L. plantarum* E282	−	−
*L. plantarum* E63	−	−
*L. plantarum* E146	−	−
*L. plantarum* E287	−	−
*L. pentosus* Ε110	−	−
*L. pentosus* 281	−	−
*L. casei Shirota* ACA-DC 6002	−	−
*L. casei* ATCC 393	−	−
*L. rhamnosus* GG ATCC 53103	−	−
*L. delbrueckii subsp. bulgaricus*	−	−

**Figure 4:**
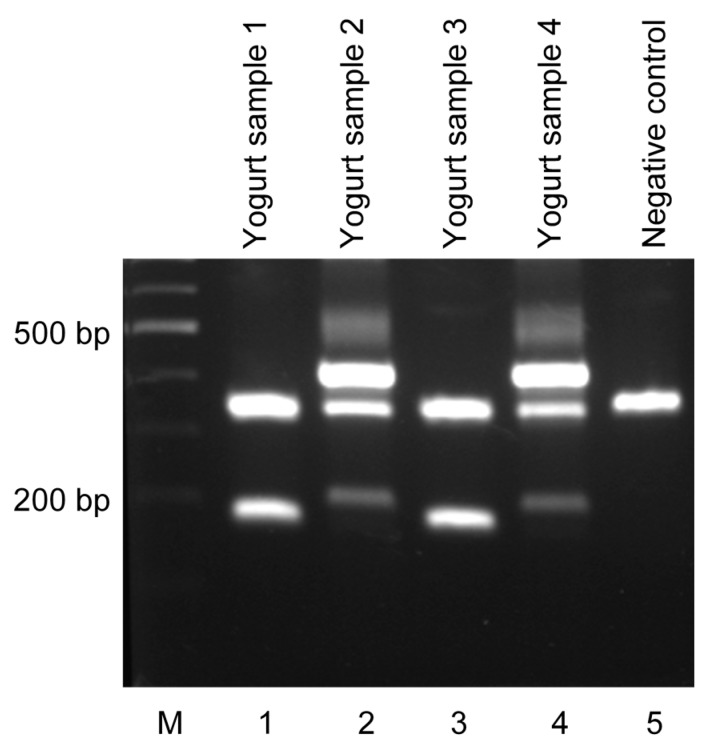
Molecular identification of *L. plantarum* 2035 or *L. plantarum* ACA-DC 2640 in food products, using the multiplex PCR assay. Lanes 1–4 correspond to yogurt samples prepared in the lab containing either *L. plantarum* 2035 (lanes 1 and 3) or *L. plantarum* ACA-DC 2640 (lanes 2 and 4); Lane 5 that corresponds to a sample prepared in the lab lacking *L. plantarum* 2035 or *L. plantarum* ACA-DC 2640 served as a negative control. The experiment was repeated three times with different yogurt preparations.

Finally, to test the applicability of our method in food products, yogurts were prepared in the lab using a commercial yogurt culture containing either *L. plantarum* 2035 (samples 1 and 3) or *L. plantarum* ACA-DC 2640 (samples 2 and 4). All samples were mixed with sterilized ¼ Ringer solution, total DNA was extracted, and multiplex PCR was implemented using the novel strain-specific primers. Two PCR products corresponding to the unique pattern of *L. plantarum* 2035 (Figure 3A) were generated for samples 1 and 3 (Figure 4). Accordingly, three PCR products, corresponding to the unique pattern of *L. plantarum* ACA-DC 2640 (Figure 3B), were generated for samples 2 and 4 (Figure 4), thus confirming the presence of the specific strains in the above samples. We are currently preparing novel dairy (cheese) and non-dairy (sausages) food products containing either *L. plantarum* 2035 or *L. plantarum* ACA-DC 2640 and are investigating their properties both *in vitro* and *in vivo*.

## 3. Experimental Section

### 3.1. Bacterial Strains and Culture Conditions

*L. plantarum* 2035 was kindly provided by K. Koutsoumanis, Laboratory of Food Microbiology and Hygiene, Dept. of Food Science & Technology, Aristotle University, Thessaloniki, Greece. *L. plantarum* ACA-DC 2640 and *L. casei Shirota* ACA-DC 6002 strains were kindly provided by E. Tsakalidou, Laboratory of Dairy Research, Agricultural University of Athens. *L. rhamnosus* GG ATCC 53103 and *L. casei* ATCC 393 strains were obtained from DSMZ (Braunschweig, Germany). All other *Lactobacillus* strains tested were obtained from the collection of the Institute of Technology of Agricultural Products, Hellenic Agricultural Organization-DEMETER, Athens, Greece. All LAB strains were grown anaerobically at 37 °C on MRS broth (Merck, Darmstadt, Germany).

### 3.2. DNA Extraction from Pure Cultures

Extraction of genomic DNA from a pure culture was performed using a DNeasy Tissue Kit (Qiagen, Hilden, Germany) according to the manufacturer’s protocol. To determine the amount of extracted DNA, the absorbance at 260 nm was measured using a UV spectrophotometer (Eppendorf, Hamburg, Germany).

### 3.3. Yogurt Production

Yogurts were manufactured by inoculating pasteurized bovine milk (100 mL) with a commercial yogurt culture CH-1 (Chr. Hansen, Hørsholm, Denmark) consisting of *Strep. salivarius* ssp. *thermophilus* and *L. delbrueckii* subsp. *bulgaricus* along with *L. plantarum* 2035 or ACA-DC 2640. Τhe initial cell counts for *L. plantarum* 2035 or ACA-DC 2640 were approximately 8–9 log cfu·g^−1^.

### 3.4. Microbial Enumeration

Lactobacilli (gram-positive, catalase-negative) counts were measured after serial diluting and plating on acidified MRS agar (Fluka, Buchs, Switzerland) at 37 °C for 48 h anaerobically (Anaerobic Jar, Anerocult C, Merck, Germany).

### 3.5. DNA Extraction from Yogurt Samples

Ten grams of a yogurt sample were blended with 90 mL of sterilized ¼ Ringers solution (Sigma-Aldrich, Gillingham, UK). Total DNA extraction from the liquid suspension was performed using a DNeasy Tissue Kit (Qiagen, Hilden, Germany) according to the manufacturer’s protocol.

### 3.6. RAPD PCR

All decamer RAPD primers used in this study were designed using the RAPD primer generator application (Available online: http://www2.uni-jena.de/biologie/mikrobio/tipps/rapd.html) (J. Wöstemeyer, Institute of General Microbiology and Microbial Genetics, Jena, Germany) and obtained from VBC-Biotech, Wien, Austria. PCR reactions were performed in a total volume of 20 μL, containing 2 units Taq DNA polymerase (Kapa Biosystems, Wilmington, NC, USA), 2 μL of 10× PCR buffer (without MgCl_2_), 200 μM each dNTPs (Kapa Biosystems), 2.5 mM MgCl_2_ (Kapa Biosystems), 100 ng template DNA, and 10 pmol of selected primer. Amplifications were performed in a Thermal Cycler (Mastercycler Eppendorf, Hamburg, Germany) under the conditions: initial denaturation at 95 °C for 5 min, followed by 30 cycles of denaturation at 95 °C for 30 s, primer annealing for 1 min at 36 °C, and primer extension for 1.5 min at 72 °C with a final extension period at 72 °C for 5 min. Then, samples were cooled to 4 °C. Aliquots (5.0 μL) of the amplified products were subjected to electrophoresis in 1.5% *w*/*v* agarose gels in TAE buffer. The gels were stained with 0.5 μg·mL^−1^ ethidium bromide, visualized under UV illumination, and photographed with a digital camera (Gel Doc EQ System, BioRad, Milan, Italy). The PCR reactions were repeated three times for each RAPD primer, and those that demonstrated reproducibility were considered for further marker isolation and cloning.

### 3.7. Cloning and Sequencing

After electrophoresis separation of the RAPD fragments, the chosen bands were excised from the gel. DNA isolation was performed using a NucleoSpin Extract II kit (MACHEREY*-*NAGEL, Düren, Germany) according to the manufacturer’s instructions. The potential strain-specific RAPD markers were cloned into the pBlueScriptSK + vector following the TA cloning protocol described by Zhou and Gomez-Sanchez [26]. Briefly, the purified DNA fragments were incubated with 2 units of Taq polymerase (Kapa Biosystems), at 72 °C for 30 min in the presence of the four dNTPs, 10 mM each dNTP (Kapa Biosystems), for the addition of 3′-A overhangs, followed by purification using a NucleoSpin Extract II kit (MACHEREY*-*NAGEL, Düren, Germany)*.* Accordingly, the plasmid was firstly digested with the restriction enzyme *Eco*RV (Fermentas, MA, USA) to generate unique blunt-ends in the multiple cloning sites of the polylinker. Then, the blunt-ended plasmid DNA was purified using a NucleoSpin Extract II kit (MACHEREY*-*NAGEL) and incubated with 5 units of Taq polymerase (Kapa Biosystems) at 72 °C for 2 h, in the presence of 100 mM TTPs (Kapa Biosystems) for the addition of 3′-T overhangs. The T-tailed plasmid DNA was then purified using a NucleoSpin Extract II kit (MACHEREY*-*NAGEL) and incubated overnight at 16 °C with 3 units of T4 DNA ligase (Fermentas) to distinguish from the vectors without T-tails. T-vectors were separated from the self-ligated and concatemerized plasmid DNA on a 1% *w*/*v* agarose gel. The extraction of the T-vector band was performed using a NucleoSpin Extract II kit (MACHEREY*-*NAGEL). The A-tailed DNA fragment was ligated into the T-vector and then transformed into *Escherichia coli* Top-10 competent cells by heat shock method according to Sambrook *et al*. [27]. The cells were then spread on Luria-Bertani (LB) medium containing appropriate ampicillin, IPTG, and X-gal and incubated at 37 °C for 16 h. Clone selection was carried out based on blue/white selection. Extraction of the recombinant plasmids was performed using a NucleoSpin Plasmid kit (MACHEREY*-*NAGEL) and checked for insert by diagnostic digest with *Hin*dIII (Fermentas) and *Eco*RI (Fermentas). The resulting clones were sent for sequencing to VBC-Biotech, Austria. The nucleotide sequence of the cloned fragment was determined from three clones. The nucleotide sequence of the cloned fragment was determined from three clones. To design the forward and reverse primers, the decamer primer was extended by a few nucleotides (8–10 bases) based on the obtained sequence. The Tm, GC, and other factors were analyzed using Primer3, primer design program (Available online: http://bioinfo.ut.ee/primer3/) and the final compatible forward and reverse primers selected for specific SCAR amplification. NCBI Primer*-*BLAST (Available online: http://www.ncbi.nlm.nih.gov/tools/primer-blast) was used to evaluate the specificity of the designed primers.

### 3.8. Multiplex PCR

Multiplex PCR reactions were performed in a total volume of 20 μL, containing 2 units of Taq DNA polymerase (Kapa Biosystems), 2 μL of 10× PCR buffer (without MgCl_2_), 200 μM each dNTPs (Kapa Biosystems), 2.5 mM MgCl_2_ (Kapa Biosystems), and 100 ng template DNA. The specific designed primers of *L. plantarum* 2035 consisted of p30F: 5′-GTGATCGCAGTTGGAAAACTG-3′ (10 pmol), and p30R: 5′-GTGATCGCAGGGAGATTATC-3′ (10 pmol) and of *L. plantarum* ACA-DC 2640 consisted of p29AF: 5′-GGGTAACGCCACAAGAAGC-3′ (8 pmol), p29BF: 5′-GTACTTGGCTAGTCGGCAG-3′ (8 pmol) and p29R: 5′-GGGTAACGCCTTGCACTTTG-3′ (16 pmol), respectively. Additionally, a set of primers consisted of Lac1F: 5′-AGCAGTAGGGAATCTTCCA-3′ and Lac1R: 5′-ATTYCACCGCTACACATG-3′ that recognize region in the *16S rRNA* gene of all lactobacilli was also included in the reactions (8 pmol of each primer) as a positive control [24]. Amplification was performed in a Thermal Cycler (Mastercycler Eppendorf, Hamburg, Germany) under the conditions: for *L. plantarum* 2035, 95 °C (2 min), followed by 24 cycles of 95 °C (30 s), 61 °C (30 s), 72 °C (60 s), followed by a final extension step at 72 °C (2 min); for *L. plantarum* ACA-DC 2640, 95 °C (2 min), followed by 22 cycles of 95 °C (30 s), 63 °C (60 s), 72 °C (60 s), followed by a final extension step at 72 °C (2 min). The PCR products were separated on 1% *w/v* agarose gels, visualized under UV illumination, and photographed with a digital camera (Gel DocEQ System, BioRad, Milan, Italy).

## 4. Conclusions

In this study, an efficient and accurate method for the detection of *Lactobacillus plantarum* strains, termed as 2035 and ACA-DC 2640, in a single reaction, was presented. It is a novel multiplex PCR assay utilizing strain-specific primers derived from RAPD analysis. The proposed methodology may be used to assess the survival and the adhesion properties of the two strains *in vivo* and characterize their probiotic character and applicability for the production of novel functional foods with potential health benefits.
